# Ligand-Based and Structure-Based Investigation for Alzheimer's Disease from Traditional Chinese Medicine

**DOI:** 10.1155/2014/364819

**Published:** 2014-05-08

**Authors:** Kai Hsin Liao, Kuen-Bao Chen, Wen-Yuan Lee, Mao-Feng Sun, Cheng-Chun Lee, Calvin Yu-Chian Chen

**Affiliations:** ^1^School of Pharmacy, China Medical University, Taichung 40402, Taiwan; ^2^School of Medicine, College of Medicine, China Medical University, Taichung 40402, Taiwan; ^3^Department of Biomedical Informatics, Asia University, Taichung 41354, Taiwan

## Abstract

Alzheimer's disease is a neurodegenerative disease that was conventionally thought to be related to the sedimentation of beta-amyloids, but drugs designed according to this hypothesis have generally failed. That FKBP52 can reduce the accumulation of tau proteins, and that Tacrolimus can reduce the pathological changes of tau proteins are new directions away from the long held amyloid-beta-centric concept. Therefore, the screening of traditional Chinese medicine compounds for those with higher affinity towards FKBP52 than Tacrolimus may be a new direction for treating Alzheimer's disease. This study utilizes ligand-based and structure-based methods as the foundation. By utilizing dock scores and the predicted pIC50 from SVM, MLR, and Bayesian Network, several TCM compounds were selected for further analysis of their protein-ligand interactions. Daphnetoxin has higher affinity and complex structure stability than Tacrolimus; Lythrancine II exhibits the most identical trends in FKBP52 interactions as Tacrolimus, and 20-O-(2′*E*,4′*E*-decadienoyl)ingenol may be further modified at its hydrocarbon chain to promote interaction with FKBP52. In addition, we observed the residue Tyr113 of FKBP52 may play a key role in protein-ligand interaction. Our results indicate that Daphnetoxin, 20-O-(2′*E*,4′*E*-decadienoyl)ingenol, and Lythrancine II may be starting points for further modification as a new type of non-amyloid-beta-centric drug for Alzheimer's disease.

## 1. Introduction

Alzheimer's disease (AD) is the most common form of neurodegenerative disease [1–3] with symptoms ranging from intellectual deterioration, cognitive impairment [4, 5] to abnormal behavior, personality changes, depression, and sleep disorders [6, 7]. Neuronal loss, senile plaques in the cerebral cortex, and neurofibrillary tangles in the temporal and frontal lobes are prominent in the brains of AD patients. Deterioration in the limbic system, including pathological changes in the hippocampus and atrophy of pyramidal cells and the amygdale, has also been reported [8, 9]. Formation of *β*-amyloid plaques [10–13] and neurofibrillary tangles due to abnormal phosphorylation of tau proteins [14] have been linked to AD.

Pioneer research has discovered that the concentration of FKBP52, a FK506-binding protein, is of high density in brain [15] and 40 times higher within the central nervous system than in the immune system [16]. FKBP52 is not only related to immune functions but is also important for CNS protective properties. Interestingly, they can easily bind with highly phosphorylated tau proteins [16], thereby reducing the accumulation of tau proteins [16–18]. FKBP52 was initially discovered as a cochaperone of steroid receptor heterocomplexes [19, 20] and is a member of the FK506-binding protein (FKBP) of immunophilins. FKBP52, which possesses a chaperone function, has a PPIase domain also called FK506-binding domain (FKBD), composed of the first 138 amino acids from the N-terminal, exhibits peptidylprolyl isomerase (PPIase) activity, and plays an important role in regulating tau proteins.

Tacrolimus (FK506) is an immunosuppressant typically used to reduce graft rejection during organ transplants [21]. Research shows that FK506 can also reduce pathological changes in tau proteins [22] and promote neuroregeneration [23, 24]. In addition, FK506 can disrupt steroid receptor heterocomplexes, leading to the release of FKBP52 from the complex and enabling it to function in the nervous system [24–26].

In recent years, most beta-amyloid-centric drug developments have been unsuccessful during Phase III of clinical trials [27, 28]. Currently, the personalized medicine and biomedicine defined an important knowledge for diagnosis and treatment analysis [29, 30], such as rare diseases [31, 32], regional disease [33], signal pathway [34–36], and gene association [37, 38]. The traditional Chinese medicine is an important medicine culture in Asia defined as a personalized medicine, system biology, and biomedicine in medicine practices widely applied as treatments for cancer [39, 40], cardiovascular disease [41, 42], diabetes [43], virus disease [44], inflammation [45], and metabolic disorders [46], indicating its large therapeutic potential for various diseases [47]. In this moment, the cloud computation and system biology could help the screening for TCM application [48, 49]. We have already designed many therapies to different targets [48, 50–57] and have successfully developed method for designing drug in disordered protein [58, 59]. The aim of this research is to screen for potential non-amyloid-beta-centric leads from traditional Chinese medicine targeting FKBP52 and to investigate its mechanisms with the hope of providing important insights for forwarding Alzheimer's disease.

## 2. Results 

### 2.1. SVM/MLR/Bayesian Network

The ten representative descriptors determined by GFA were* ES_Count_aaCH*,* ES_Count_sOH*,* ES_Sum_aaCH*,* ES_Sum_sssN*,* Num_Rings6*,* Molecular_PolarSurfaceArea*,* CHI_2*,* Jurs_TPSA*,* Minimized_Energy*, and* Shadow_Ylength*, suggesting certain relationships between electrotopological properties and polar surface area of ligands with bioactivity (Table S1).

The GFA model with the highest correlation between experimental and predicted pIC_50_ values (*R* ^2^ = 0.9402) was

(1) GFATempModel_1 =14.911+80.578×ES_Count_aaCH  −27.224×ES_Count_sOH−44.517×ES_Sum_aaCH  +16.799×ES_Sum_sssN−27.187×Num_Rings6  +0.90601×Molecular_PolarSurfaceArea  −9.6615×CHI_2−0.13155×Jurs_TPSA  −1.1131 Minimized_Energy  +13.232×Shadow_Ylength.

**Figure 1:**
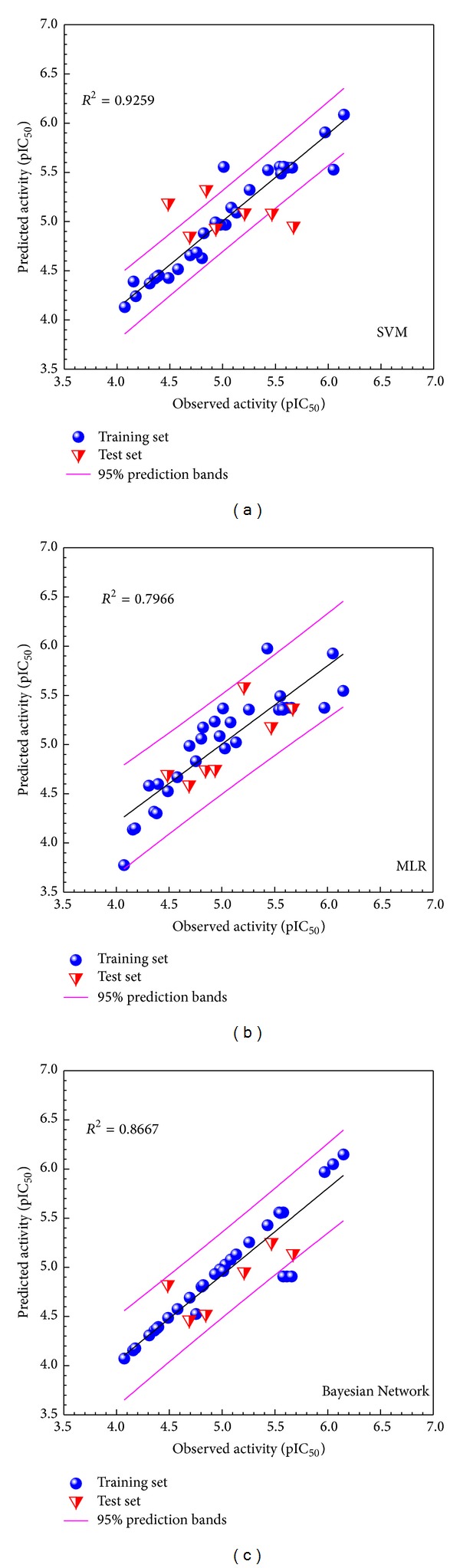
Correlation plot of observed bioactivity versus predicted bioactivities generated by different models: (a) SVM, (b) MLR, and (c) Bayesian Network. Training and test sets are represented by blue circles and red triangles, respectively.

Correlation of experimental and predicted pIC_50_ using the constructed SVM, MLR, and Bayesian Network models is illustrated in Figure 1. *R* ^2^ values indicate good prediction accuracy of the constructed models.  Table 1 summarizes bioactivities of the top ten ligands and Tacrolimus predicted by the three constructed models.

### 2.2. Docking Analysis

**Figure 2:**
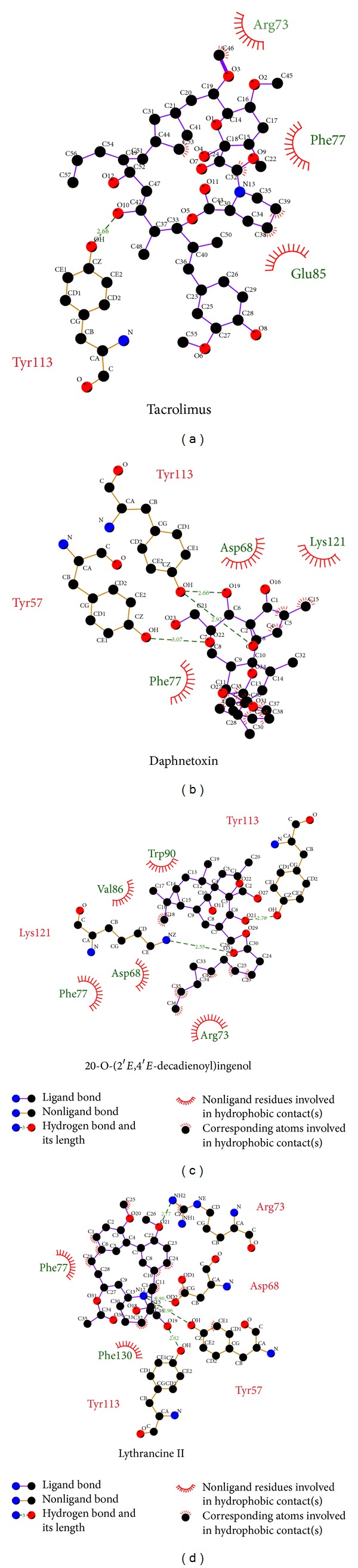
Hydrophobic interactions and H-bonds illustrated through LigPlot for (a) Tacrolimus, (b) Daphnetoxin, (c) 20-O-(2′*E*,4′*E*-decadienoyl)ingenol, and (d) Lythrancine II.

Summary of docking interactions between FKBP52 and the selected ligands is listed in Table 2. Tacrolimus interacted with FKBP52 via a single H-bond at Tyr113 and three hydrophobic interactions. Higher amounts of total interactions were recorded for TCM candidates. With regard to individual residues, interaction percentages suggest the importance of Arg73, Phe77, and Tyr113 for ligand binding. Interactions with Arg73 were ligand-dependent, whereas Phe77 and Tyr113 were mutual interaction sites for hydrophobic interactions and H-bonds, respectively. Cross comparison reveals that TCM candidates form interactions with all Tacrolimus-interacting residues except Glu85. In addition, each TCM candidate is anchored by interactions with residues not recorded for Tacrolimus. The higher number of interactions observed for TCM candidates might be associated with higher dock scores and suggests higher binding affinity to FKBP52 than to Tacrolimus.  Figure 2 was demonstrated by Ligplot, showing the hydrophobic interactions and hydrogen bonds between control compound and TCM candidates. Except for the H-bond between residue Tyr57 and Daphnetoxin, the results were the same for docking analysis.

### 2.3. Candidate Selection

**Figure 3:**
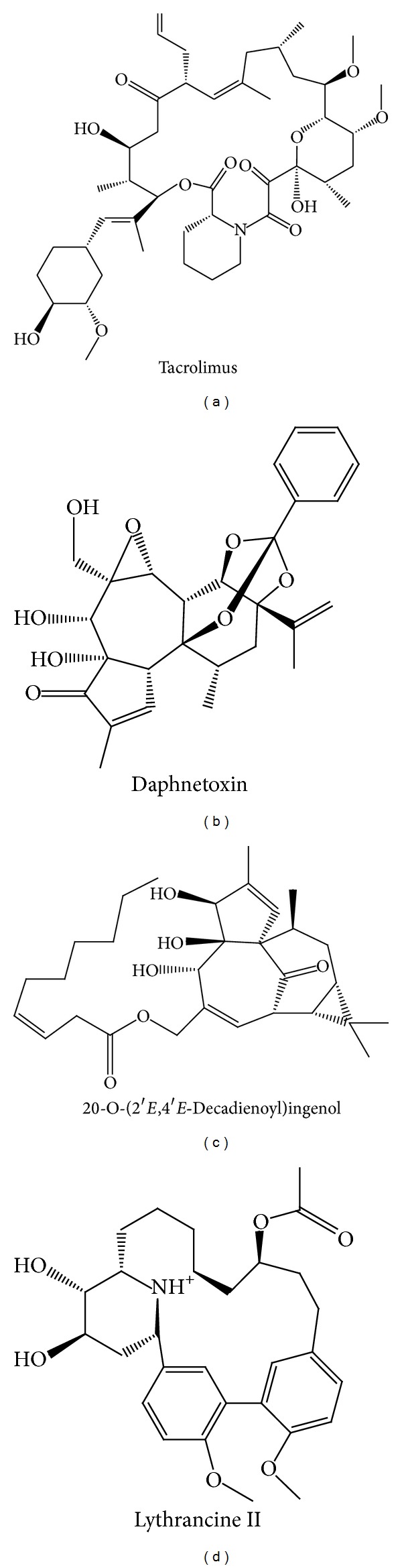
Chemical scaffold of (a) Tacrolimus, (b) Daphnetoxin, (c) 20-O-(2′*E*,4′*E*-decadienoyl)ingenol, and (d) Lythrancine II.

Docking scores, predicted bioactivities, and corresponding consensus scores are detailed in Table 1, respectively. In general, candidates with higher dock scores than Tacrolimus were also predicted to have similar if not better bioactivities. Daphnetoxin (TCM origin:* Daphne giraldii *Nitsche), 20-O-(2′*E*,4′*E*-decadienoyl)ingenol (TCM origin:* Euphorbia kansui*), and Lythrancine II (TCM origin:* Lythrum salicaria*) were selected as research candidates based on the consensus voting system (Table 1). Structural scaffolds of Tacrolimus and the selected candidates are illustrated in Figure 3.

### 2.4. Molecular Dynamics Simulation

#### 2.4.1. Stability Profile Analysis

**Figure 4:**
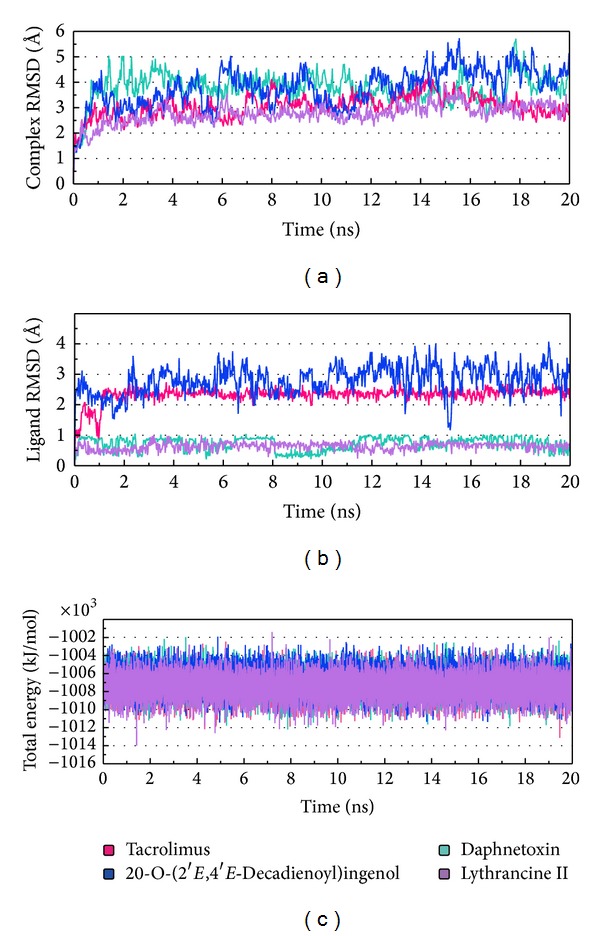
MD trajectories for (a) protein-ligand complex RMSD, (b) ligand RMSD, and (c) total energy. Trajectories for Tacrolimus, Daphnetoxin, 20-O-(2′*E*,4′*E*-decadienoyl)ingenol, and Lythrancine II are shown in pink, mint, blue, and violet, respectively.

Figure 4 illustrates protein-ligand complex stability in terms of RMSD and total energy. In general, the complex RMSD of Daphnetoxin and 20-O-(2′*E*,4′*E*-decadienoyl)ingenol was higher (1.5 $\overset{´}{Å}$–5.5 $\overset{´}{Å}$) and showed larger fluctuation than that of Tacrolimus and Lythrancine II. RMSD trajectories of FKBP52 complexed with Tacrolimus or Lythrancine II were similar, ranging between 1.5 $\overset{´}{Å}$ and 4 $\overset{´}{Å}$. With the exception of 20-O-(2′*E*,4′*E*-decadienoyl)ingenol, ligands reached dynamic equilibrium with small fluctuations during MD simulation. Notwithstanding differences in complex or ligand RMSD, all complexes exhibited stable complex energy trajectories between −1,010,000 kJ/mol and −1,004,000 kJ/mol during MD.

### 2.5. RMS Fluctuation (RMSF) of Protein Residues

**Figure 5:**
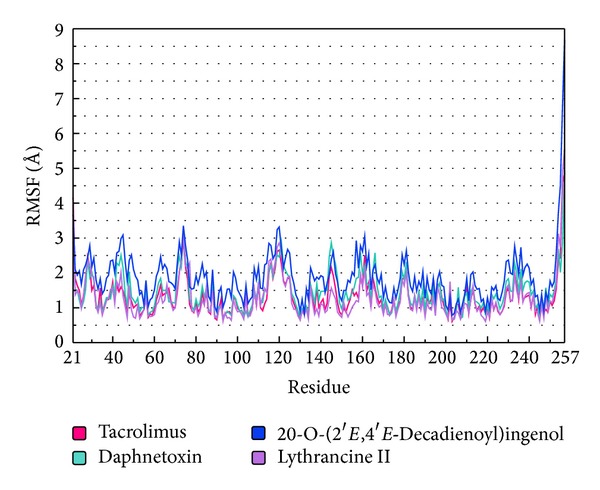
Analysis of RMS Fluctuation (RMSF) trajectories generated by Gromacs. Trajectories for Tacrolimus, Daphnetoxin, 20-O-(2′*E*,4′*E*-decadienoyl)ingenol, and Lythrancine II are shown in pink, mint, blue, and violet, respectively.

Residues contributing to complex structural fluctuations can be assessed by root mean square fluctuations (RMSFs) of each residue (Figure 5). Tacrolimus and Lythrancine II have lower RMSFs and similar trends. 20-O-(2′*E*,4′*E*-Decadienoyl)ingenol has the highest RMSF of the four, implying greater fluctuations. Daphnetoxin falls between Tacrolimus, Lythrancine II, and 20-O-(2′*E*,4′*E*-decadienoyl)ingenol. Analysis of the RMSF values shows that great differences among the protein complexes were observed at Tyr113, a residue near the binding site.

### 2.6. H-Bond Network during MD Simulation

**Figure 6:**
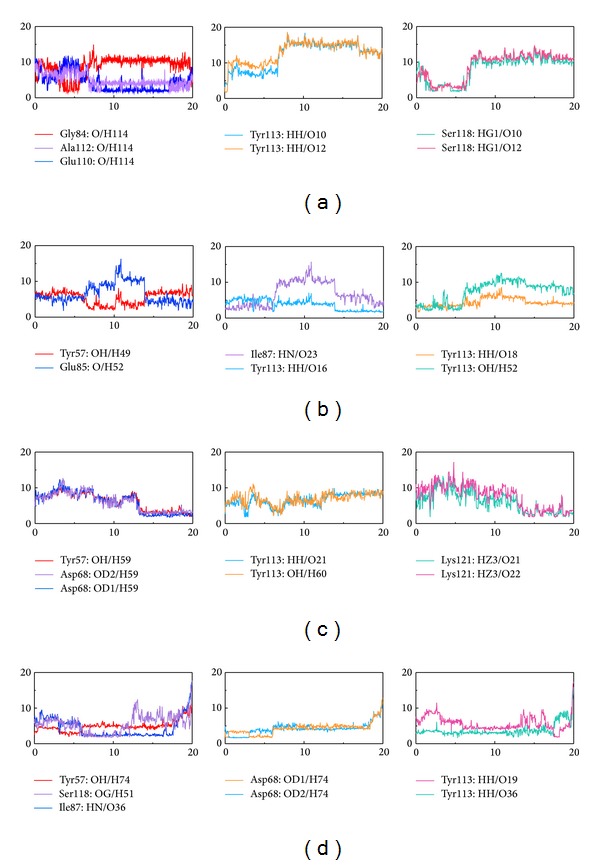
Distance of hydrogen bonds (Å) between FKBP52 and control and TCM candidates versus MD simulation time. (a) Tacrolimus, (b) Daphnetoxin, (c) 20-O-(2′*E*,4′*E*-decadienoyl)ingenol, and (d) Lythrancine II. Horizontal axis represents MD simulation time (ns), and vertical axis represents distance of hydrogen bonds (Å).

Next we investigated formation and stability of H-bonds under dynamic conditions. Individual occupancies of detected H-bonds per ligand are detailed in Table S2. H-bond trajectories depicting time-dependent bond distance variations are illustrated in Figure 6. Since optimum H-bonds are formed between 2.3 and 3.2 Å and most bond distances formed between Tacrolimus and FKBP52 residues exceed this distance, we conclude that, during MD, the protein-ligand complex stability was maintained by interactions with Glu110 and Ala 112.

Daphnetoxin forms several discontinuous H-bonds with residues of FKBP52. However, the H-bond occupancies at Tyr113 with O16, O18, and H52 of Daphnetoxin are 31.47%, 2.00%, and 9.89%, respectively. It suggests that the Tyr113 might play an important role in protein-ligand complex stability.

20-O-(2′*E*,4′*E*-Decadienoyl)ingenol and the residues of FKBP52 form some unstable H-bonds. Tyr113, O21 and H60 form only transient H-bonds during MD, whereas Asp68 and Lys121 form a stable H-bond with 20-O-(2′*E*,4′*E*-decadienoyl)ingenol from 13 to 20 ns.

Lythrancine II and residues of FKBP52 form two stable H-bonds: Ile87 forms a stable H-bond at O36 from 6 to 17.5 ns. Tyr113 and O19 on Lythrancine II form a stable H-bond from 0 to 17.5 ns. The two residues are important for protein-ligand complex stability.

### 2.7. Torsion MD Simulation

**Figure 7:**
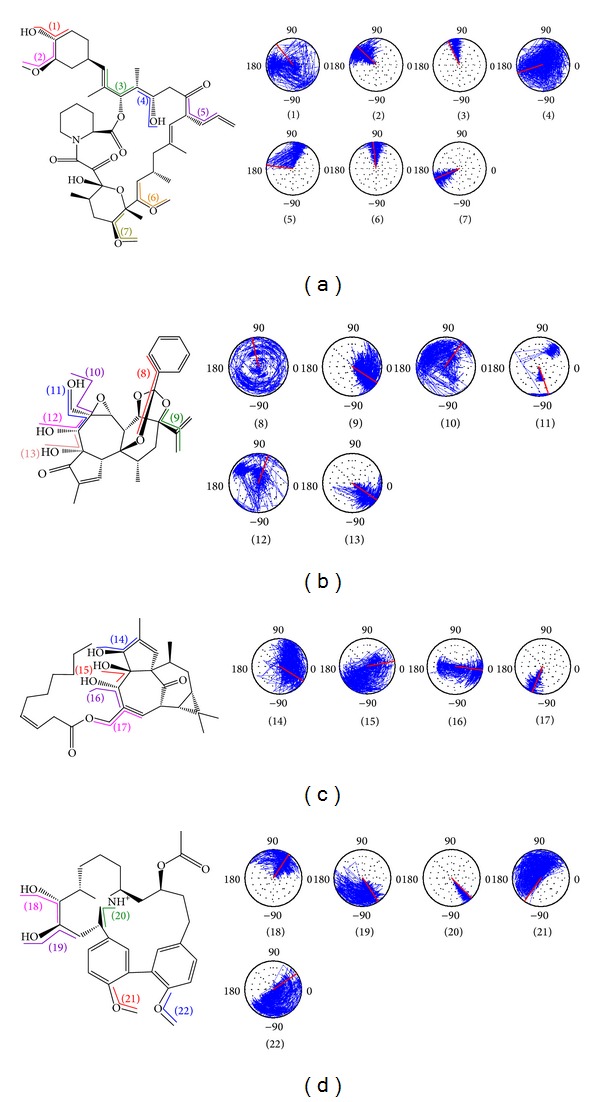
Torsion angles of control and TCM candidates in FKBP52 complex. (a) Tacrolimus, (b) Daphnetoxin, (c) 20-O-(2′*E*,4′*E*-decadienoyl)ingenol, and (d) Lythrancine II.

Analysis of torsion helps us understand the stability of ligand and protein binding (Figure 7, Figure S1; see Supplementary Material available online at http://dx.doi.org/10.1155/2014/364819). Torsion (A1) of Tacrolimus changes from significant unstable rotations (0–7.5 ns) to stable rotations (7.5–17 ns), and change to unstable rotations after 17 ns. Comparing to H-bond simulation results, we can assume that Glu110 forms a more stable H-bond. Torsion (A4) is more stable from 2 to 6 ns. During this period, H-bond simulation shows the formation of an H-bond between FKBP52 and Ser118. Torsion (A5) shows some changes, but the small torsion fluctuations at (A2), (A3), (A6), and (A7) show that they have higher stability. Torsion (B8) of Daphnetoxin shows transient stability. Torsion (B9) is stable with small fluctuations. Torsion (B10) has smaller torsion fluctuations from 0 to 10 ns; torsion (B11) also has very small changes. During this period, they separately form H-bonds with Glu85 and Ile87 on FKBP52. Torsion (B12) has small fluctuations from 8 to 18 ns, and torsion (B13) is relatively stable; these locations form H-bonds with Tyr57 and Tyr113. Violent fluctuations at (C14) were observed for 20-O-(2′*E*,4′*E*-decadienoyl)ingenol, a phenomenon probably due to the lack of stabilizing bonds with FKBP52. Torsions (C15) and (C16) only form short lived H-bonds which could be attributed to the large torsion changes. Torsion (C17) is relatively stable. With the exception of isolated large fluctuations, torsion (D18) is relatively stable, probably due to the formation of H-bond with Ser118. Torsion (D19) is relatively stable up until 12.5 ns when increased fluctuation was observed. Torsion (D20) is stable, most likely due to the H-bonds formed between Lythrancine II and Tyr57 and Asp68. Torsions (D21) and (D22) had large fluctuations.

### 2.8. DSSP MD Simulation

**Figure 8:**
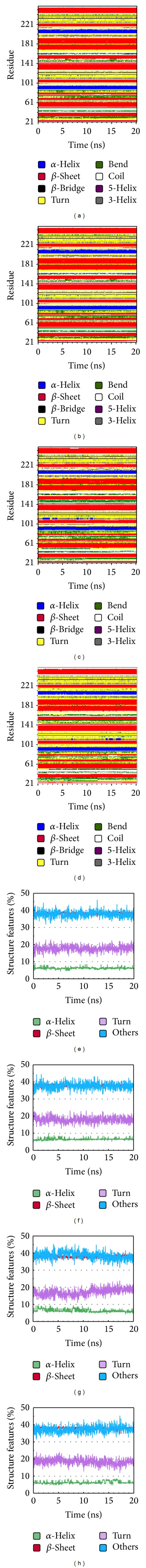
Secondary structure changes observed during the 20 ns MD simulation for (a) Tacrolimus, (b) Daphnetoxin, (c) 20-O-(2′*E*,4′*E*-decadienoyl)ingenol, and (d) Lythrancine II. Their percentages of *α*-helix, *β*-sheet, turn, and others are shown in (e), (f), (g), and (h), respectively.

Protein functionality is affected by the tertiary structure formed by the secondary structures. The conformation changes of protein secondary structures for each time frame can be computed by the DSSP program. Changes in the secondary protein structure when Tacrolimus, Daphnetoxin, 20-O-(2′*E*,4′*E*-decadienoyl)ingenol, or Lythrancine II is bound to FKBP52 are illustrated in Figure 8. Tacrolimus, Daphnetoxin, and Lythrancine II have an *α*-helix composition of approximately 6%, but that of 20-O-(2′*E*,4′*E*-decadienoyl)ingenol ranges between 6 and 10%. Compositions of *β*-sheets and turns in the four complexes are approximately 38-39% and 15–20%, but an increase in turn structures was observed in 20-O-(2′*E*,4′*E*-decadienoyl)ingenol from 12 ns. From Figure 8 we can observe more significant changes at residue 51. In Tacrolimus, residue 51 is a bend, but in Daphnetoxin, it turns into a turn at 0-1 ns and 12–18 ns. Similarly in 20-O-(2′*E*,4′*E*-decadienoyl)ingenol, the residue becomes a turn after 1 ns and after 11 ns. In Lythrancine II, it first appears as a turn from 0 to 2 ns, then turns to bend, and then fluctuates between bends and turns. Comparisons to RMSFs show that RMSF of residue 51 is also larger.

### 2.9. Solvent Accessible Surface Area (SASA) in MD Simulation

**Figure 9:**
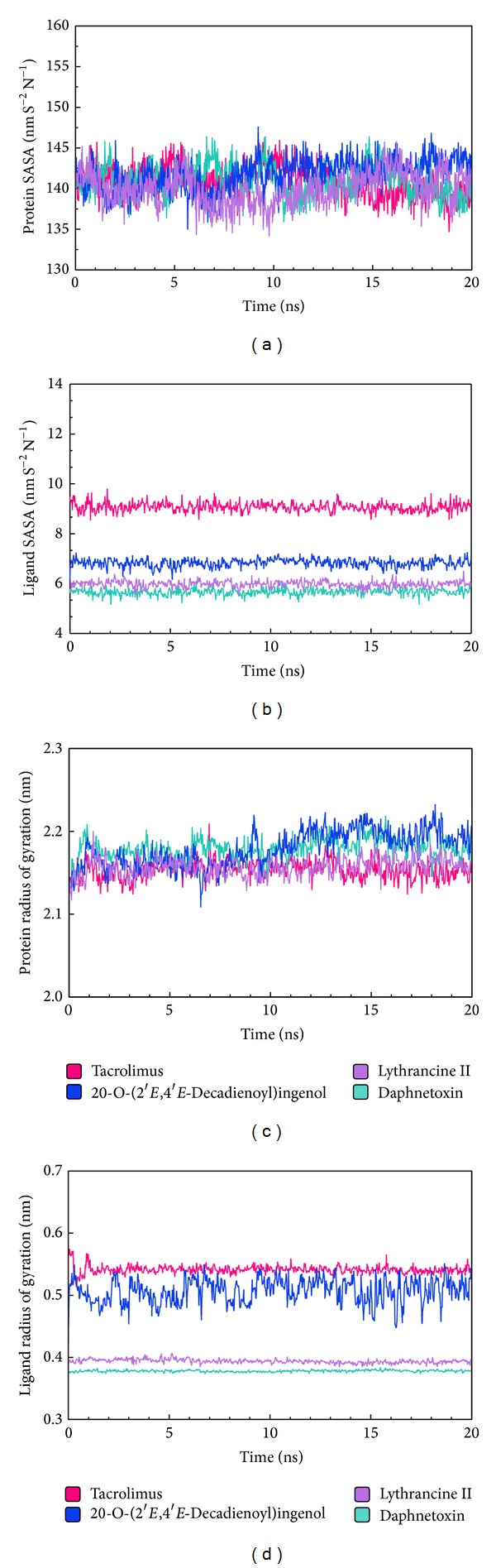
Analysis of MD trajectories generated by Gromacs: (a) solvent accessible surface area of protein, (b) solvent accessible surface area of ligands, (c) radius of gyration of protein, (d) and radius of gyration of ligands. Trajectories for Tacrolimus, Daphnetoxin, 20-O-(2′*E*,4′*E*-decadienoyl)ingenol, and Lythrancine II are shown in pink, mint, blue, and violet, respectively.

Solvent accessible surface area (SASA) analysis measures the interaction between complexes and solvents. SASAs of the ligand-protein complexes are 140.788 nm/NS^2^, 141.028 nm/NS^2^, 141.745 nm/NS^2^, and 140.004 nm/NS^2^ for Tacrolimus, Daphnetoxin, 20-O-(2′*E*,4′*E*-decadienoyl)ingenol, and Lythrancine II, respectively. Average of the ligand SASAs is 9.068 nm/NS^2^, 5.669 nm/NS^2^, 6.827 nm/NS^2^, and 5.978 nm/NS^2^, respectively. According to the results of proteins and ligands, both the values of 20-O-(2′*E*,4′*E*-decadienoyl)ingenol are the greatest, suggesting that it should be accessible for solvents and have more interaction with solvents. In addition, we observed that the SASA values for the four protein complexes (Figure 9(a)) and the ligands (Figure 9(b)) during MD is relatively stable, indicating no significant changes in the protein structure.

### 2.10. Radius of Gyration (Rg) in MD Simulation

Radius of gyration (Rg) enables one to assess the compactness changes of a ligand-protein complex. Average and maximum values for Tacrolimus, Daphnetoxin, 20-O-(2′*E*,4′*E*-decadienoyl)ingenol, and Lythrancine II are listed in Table 3. No significant changes were observed for Rg_complex_ (Figure 9(c)) and Rg_ligand_ (Figure 9(d)), implying a sustained stability and compactness of the complexes. However, fluctuations in Rg_complex_ and Rg_ligand_ were recorded for 20-O-(2′*E*,4′*E*-decadienoyl)ingenol, suggesting a loss of compactness for its complex. Comparing to H-bond simulation results in Figure 6, the Rg_complex_ of Tacrolimus presents maxima at 7 ns, and Gly84, Tyr113, and Ser118 trajectories also have the same trade in Figure 6(a). The Rg_complex_ of Daphnetoxin presents maxima at 3.5 ns, and Tyr113 with H52 at 3.5 ns also presents maxima. Moreover, the Rg_complex_ at 6 ns and 11 to 14 ns have higher values, and they correspond to Ile87, Glu85, and Tyr113 trajectories in Figure 6(b). The minima of Rg_complex_ at 6.5 ns in 20-O-(2′*E*,4′*E*-decadienoyl)ingenol, similar to that of Tyr113, Lys121 trajectories (Figure 6(c)). In addition, the Rg_complex_ presents maxima at 9 ns, and Tyr113 trajectory matches it. The Rg_complex_ of Lythrancine II presents two maxima at 11 ns and 15 ns, corresponding to Asp68 and Ser118 trajectories and Tyr113 trajectory, respectively (Figure 6(d)). In conclusion, when Rg_complex_ is higher, the compactness of ligand-protein complex is lower causing the interactions between ligand and protein to be more weak.

### 2.11. Cluster MD Simulation

**Figure 10:**
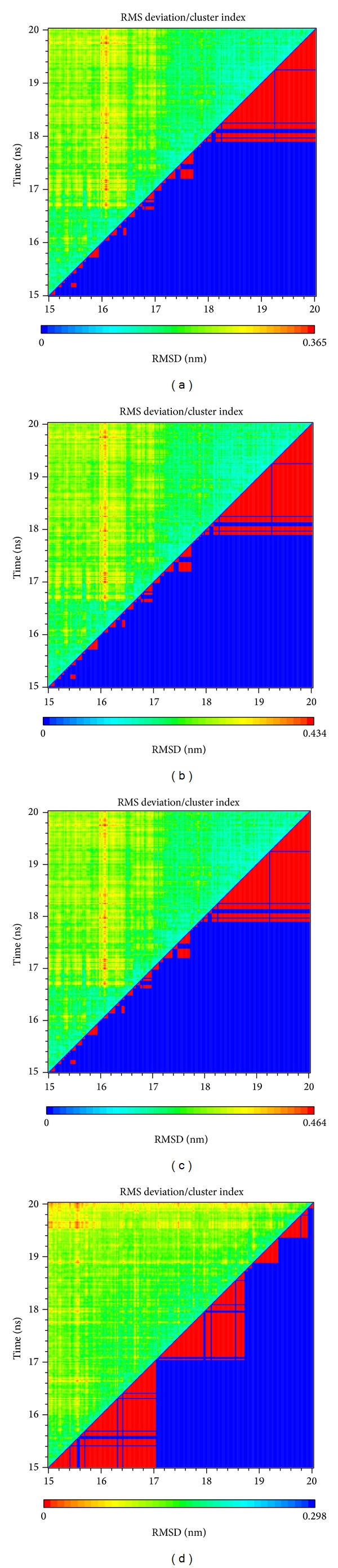
Cluster index and RMSD between amino acid: (a) Tacrolimus, (b) Daphnetoxin, (c) 20-O-(2′*E*,4′*E*-decadienoyl)ingenol, and (d) Lythrancine II.

MD simulations from 15 to 20 ns were used for cluster analysis (Figure 10 and Figure S2) to find the conformation with the highest frequency during the end of MD. According to our analysis results, the representative conformation for each protein-ligand complex was represented by the conformations at 19.02 ns for Tacrolimus, 19.32 ns for Daphnetoxin, 19.58 ns for 20-O-(2′*E*,4′*E*-decadienoyl)ingenol, and 19.72 ns for Lythrancine II.

**Figure 11:**
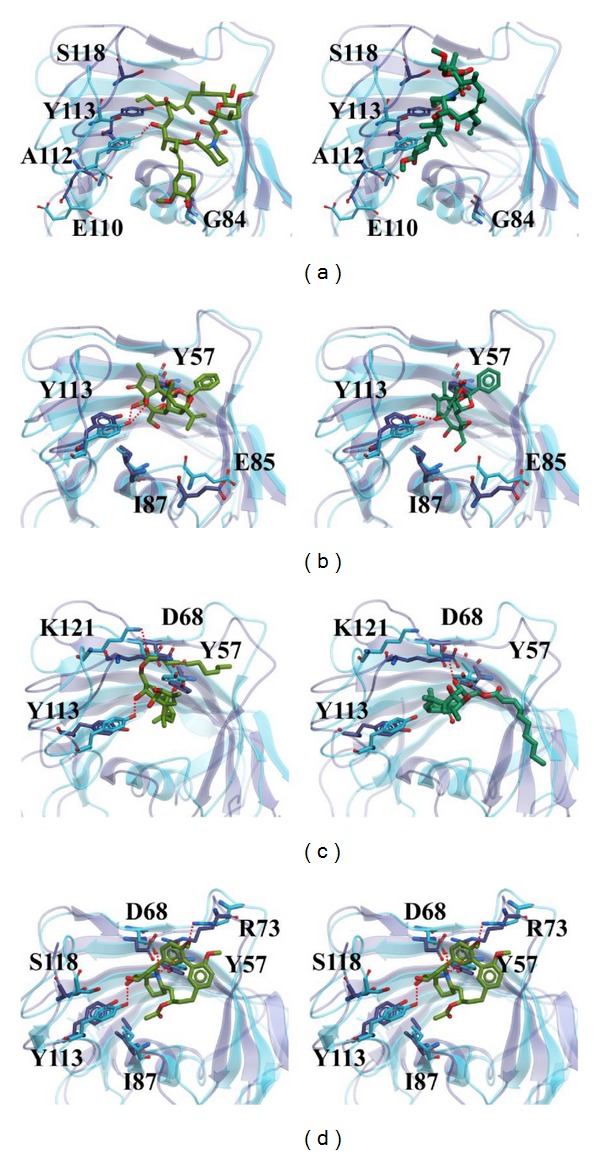
The conformation of protein-ligand complex at 0 ns of MD simulation on the left side compared to the conformation at specific time of MD simulation on the right side. The conformations of (a) Tacrolimus, (b) Daphnetoxin, (c) 20-O-(2′*E*,4′*E*-decadienoyl)ingenol, and (d) Lythrancine II are at 19.02 ns, 19.32 ns, 19.58 ns, and 19.72 ns during MD simulation, respectively. The protein structure and residues shown in blue and the ligand shown in light green indicate the conformation at 0 ns. On the other hand, the protein structure and residues shown in violet and the ligand shown in deep green indicate the conformation at specific time of MD simulation.

Comparing the conformation at 0 ns and the representative conformation for each complex is shown in Figure 11. Tacrolimus initially form an H-bond with Tyr113, but this H-bond is lost following stabilization. Moreover, the movement of Tacrolimus into the interior of the protein complex (Figure 11(a)) and the higher ligand RMSD (Figure 4) were observed. Therefore, the significant change of Tacrolimus might cause the H-bond to disappear and impact the interaction between Tacrolimus and FKBP52 deeply. The complex RMSD of Daphnetoxin is higher, but the ligand RMSD reach dynamic equilibrium with small fluctuations (Figure 4), it might cause Daphnetoxin to form two H-bonds with Tyr113, and this was maintained throughout MD (Figure 11(b)). The conformation of 20-O-(2′*E*,4′*E*-decadienoyl)ingenol and protein shows great differences between 0 ns and 19.58 ns at MD simulation. Especially, the position and the side chain of ligand have obvious changes. Comparing to Figure 4, its complex RMSD and ligand RMSD are higher and show larger fluctuation. As a result, 20-O-(2′*E*,4′*E*-decadienoyl)ingenol forms H-bonds with Tyr113 and Lys121 at the beginning of MD, but only the bond with Lys121 was maintained following stabilization (Figure 11(c)). Lythrancine II forms H-bonds with Asp68, Arg73, and Tyr113 at the beginning of MD, but these bonds are lost after stabilization, even though its complex RMSD and ligand RMSD are not higher. It might be attributed to the huge conformation change of the ligand. Similar to Tacrolimus, Lythrancine II was observed to be more embedded in the interior of the protein complex (Figure 11(d)).

## 3. Discussion

Discrepancies between docking and MD simulation results were observed. During docking, Tacrolimus, Daphnetoxin, 20-O-(2′*E*,4′*E*-decadienoyl)ingenol, and Lythrancine II were predicted to form H-bonds with Tyr113 of FKBP52. Nonetheless, occupancies of the H-bond with Tyr113 varied greatly. Similarly, RMSF also shows that Tyr113 has great differences among the four complexes. In Table S2, we can see that the occupancies of Tyr113 in the Tacrolimus complex are only 0.3% and 1.6%. The occupancies of Tyr113 in the Daphnetoxin complex are 31.47% and 2.00%; therefore the H-bond can still be observed when stabilized. The occupancies of Tyr113 in the 20-O-(2′*E*,4′*E*-decadienoyl)ingenol complex are only 1.8% and 0.6%. But the bonds with Lys121 were observed during docking and were maintained during MD simulation with occupancies of 9.09% and 8.99%. The occupancies of Tyr113 with Lythrancine II complex are 9.29% and 3.5%, but H-bonds were not observed in the stabilized complex.

From other MD simulation analysis we can make the following summarizations. Despite the larger protein complex RMSD of Daphnetoxin compared to Tacrolimus, the H-bond with Tyr113 was presented during both docking and MD simulation, indicating a better affinity towards FKBP52 than Tacrolimus. Summarizing the results from RMSF, SASA, and Rg, the protein complex of Daphnetoxin is stable during MD simulation. 20-O-(2′*E*,4′*E*-Decadienoyl)ingenol has higher complex and ligand RMSDs than Tacrolimus and also has more prominent changes in secondary structure changes, RMSF, and Rg. This could be related to the hydrophobic hydrocarbon backbone in its structure. However, 20-O-(2′*E*,4′*E*-decadienoyl)ingenol forms more hydrophobic interactions than Tacrolimus and is able to maintain the H-bond with Lys121 following stabilization, suggesting affinity with FKBP52. In addition, 20-O-(2′E,4′E-decadienoyl)ingenol has maximum values of SASA analysis of proteins and ligands, indicating that it should be accessible for solvents and have more interaction with solvents. Though Lythrancine II do not have H-bonds in the stabilized MD simulation conformation, its complex RMSD, ligand RMSD, RMSF, and Rg trends are most similar to Tacrolimus. In addition, movement of Lythrancine II towards the protein interior is also reminiscent of Tacrolimus. Therefore, Lythrancine II shows the most similar ligand-protein conformation with Tacrolimus. From this study, we can also see the importance of MD simulation in computer-aided drug designs.

## 4. Conclusion

We aimed to utilize structure-based and ligand-based methods to screen for high affinity lead compounds for FKBP52 as alternatives for Tacrolimus to develop better non-amyloid-beta-centric therapies for Alzheimer's disease. Utilizing docking and highly reliable QSAR models, Daphnetoxin, 20-O-(2′*E*,4′*E*-decadienoyl)ingenol, and Lythrancine II were selected as potential candidates. Docking, Ligplot, and MD show that Daphnetoxin and Lythrancine II both have better affinity to FKBP52 than Tacrolimus. For 20-O-(2′*E*,4′*E*-decadienoyl)ingenol, modifying its long chain hydrocarbon could improve interaction with FKBP52. Based on these results, there is possibility to utilize Daphnetoxin, 20-O-(2′*E*,4′*E*-decadienoyl)ingenol, and Lythrancine II as backbone structures for modification as a new approach for designing Alzheimer's disease.

## 5. Material and Methods

### 5.1. Data Collection

The FKBP52 crystal structure (PDB ID: 1Q1C) was downloaded from Protein Data Bank [60] and corrected for H-atoms using the* Prepare Ligands* module in Discovery Studio Client v2.5 (DS2.5; Accelrys Inc., San Diego, CA). Ligand structures and activity data from Gopalakrishnan's study [61] were used to construct quantitative structure-activity relationship (QSAR) prediction models for FKBP52. TCM ligands used for virtual screening were downloaded from TCM Database@Taiwan [62]. Tacrolimus (FK506), the clinically used immunosuppressant, was used as the control.

### 5.2. Molecular Docking

Prior to virtual screening,* Change Ionization* in the* Prepare Ligands* module (DS 2.5) was applied to adjust ionization states of the downloaded ligands. Downloaded ligands and Tacrolimus were docked to the PPIase domain of FKBP52 under a forcefield of Chemistry at HARvard Molecular Mechanics (CHARMm) using the* LigandFit* module. Ligands were evaluated based on structural compatibility to the PPIase domain and dock score was selected as the scoring function.

### 5.3. Support Vector Machine (SVM) and Multiple Linear Regression (MLR) Prediction Models

The 37 ligands [61] were randomly divided into a training set of 30 compounds and a test set of 7 compounds. Molecular properties of each ligand were calculated with* Calculate Molecular Properties* and the ten representative descriptors most related to bioactivity were determined by Genetic Function Approximation (GFA). Different prediction models were constructed with the representative descriptors and the strength of each model ranked by a square correlation coefficient (*R* ^2^). Descriptors in the highest *R* ^2^ model were used to construct a nonlinear SVM model with LibSVM and a linear MLR model with MATLAB. The constructed models were validated with the test sets and applied to predict bioactivity of selected TCM candidates.

### 5.4. Bayesian Network

Training/test set groups and representative descriptors determined by GFA were also used to construct the Bayesian Network model. According to distribution characteristics, descriptors and pIC_50_ were discretized into a maximum of five categories. Linear regression analysis for each pIC_50_ category in the training dataset was then applied. Banjo [63] was used to discover relationships among the representative descriptors and pIC_50_ values. The algorithm for predicting pIC_50_ was written in MATLAB codes integrating Banjo and Bayes Net Toolbox (BNT; https://code.google.com/p/bnt/). Following validation, the algorithm was applied to predict bioactivity of selected TCM candidates.

### 5.5. Candidate Selection Criteria

All TCM ligands with dock scores lower than Tacrolimus were eliminated. Next, a consensus voting system was used for candidate selection. The three highest scoring candidates in dock score, SVM, or MLR were given a score of “1”; candidates with the three highest Bayesian Network scores were given a score of “2.” Sum of scores was calculated and the three ligands with the highest scores were selected as candidates for further analysis.

### 5.6. Molecular Dynamics (MD) Simulation

Candidates and Tacrolimus were prepared with SwissParam (http://swissparam.ch/) [64] prior to MD. MD simulation was conducted with GROMACS 4.0.7 under the forcefield of CHARMm27. Distance of protein to box boundaries was 1.2 nm; solvate TIP3P water model was then added to the system. Complex charges were neutralized with sodium and chloride ions using 0.145 M salt model. Simulation was conducted at 310 K under a pressure of 1 bar. Each complex was minimized with 5,000 steps of Steepest Descent, and the final minimized structure was used as the initial structure for MD simulation. Electrostatic interactions were calculated with Particle-Mesh-Ewald (PME) [65]. Equilibration protocol was used to restrain and relax protein-ligand position; first-order kinetics was started from 300 K. Minimized system was used to simulate a five-thousand ps configuration production. MD simulation was conducted for 20 ns with time steps of 2 fs under PME.

## Supplementary Material

Table S1. Descriptions of representative descriptors associated with bioactivity determined by GFA.
Table S2. H-bond interactions of FKBP52 with TCM candidates and Tacrolimus in MD simulation.
Figure S1. Details about torsion angles of control and TCM candidates in FKBP52 complex. (A) Tacrolimus, (B) Daphnetoxin, (C) 20-O-(2*´*E,4*´*E-decadienoyl)ingenol, and (D) Lythrancine II. (gray(X):original torsion anglesred(Y): if X<0,Y=X+360, blue(Z)=Y(i+1)-Y)
Figure S2. Distance matrices depicting the smallest distance between residue pairs. (A) Tacrolimus, (B) Daphnetoxin, (C) 20-O-(2*´*E,4*´*E-decadienoyl)ingenol, and (D) Lythrancine II.

## Figures and Tables

**Table 1:** Docking scores, bioactivity predictions, and consensus voting^a^ of the TCM candidates and control.

Name	SVM	MLR	Bayesian Network	Dock score	Sum of scores
Lathyranoic acid A	4.81 (0)	5.12 (0)	5.45 (0)	86.20 (1)	1
Daphnetoxin	**5.09 (0)**	**8.89 (1)**	**7.46 (2)**	**84.88 (1)**	**4**
Aurantiamide	4.89 (0)	6.42 (0)	5.43 (0)	83.89 (1)	1
(6aR,11aR)-9,10-Dimethoxypterocarpan-3-O-beta-D-glucoside	5.00 (0)	8.33 (1)	5.56 (0)	83.50 (0)	1
Picrasidine M	5.10 (1)	7.51 (0)	5.65 (0)	69.60 (0)	1
12-O-Acetylphorbol-13-tigliate	4.91 (0)	7.40 (0)	6.15 (0)	68.88 (0)	0
20-O-(2′*E*,4′*E*-Decadienoyl)ingenol	**4.91 (0)**	**6.87 (0)**	**6.35 (2)**	**68.52 (0)**	**2**
Howiinol A II	5.24 (1)	6.87 (0)	5.40 (0)	68.11 (0)	1
Moellendorffiline	5.15 (1)	6.64 (0)	5.98 (0)	63.24 (0)	1
Lythrancine II	**5.03 (0)**	**8.92 (1)**	**6.74 (2)**	**62.03 (0)**	**3**

Tacrolimus*	**5.11 **	**6.25 **	**5.33 **	**58.10 **	**—**

*Control. Consensus voting not applied to control.

^a^Voting scores are given in parenthesis.

**Table 2:** Protein-ligand interactions recorded during docking.

	Tyr57	Asp68	Arg73	Phe77	Glu85	Val86	Trp90	Tyr113	Lys121	Phe130	Total^a^
Tacrolimus	—	—	Hb	Hb	Hb	—	—	H	—	—	**4**
Daphnetoxin	—	Hb	Pi	Hb	—	—	—	H	Hb	—	**5**
20-O-(2′*E*,4′*E*-Decadienoyl)ingenol	—	Hb	Hb	Hb	—	Hb	Hb	H	H	—	**7**
Lythrancine II	H	H	H	Pi/Hb	—	—	—	H	—	Hb	**7**

Interaction percentage^b^	25%	75%	100%	100%	25%	25%	25%	100%	50%	25%	

^a^Sum of FKBP52 residues interacting with the specified ligand.

^b^Percentage of ligands which interacts with the specified residue.

Hb: hydrophobic interaction.

H: hydrogen bond.

Pi: *π*-*π* interaction.

**Table 3:** Radius of gyration (Rg) calculated for the control and TCM candidates.

	Radius of gyration (Rg; nm)							
	Tacrolimus		Daphnetoxin		20-O-(2′*E*,4′*E*-Decadienoyl)ingenol		Lythrancine II	
	Average	Maximum	Average	Maximum	Average	Maximum	Average	Maximum
Protein	2.154	2.210	2.179	2.219	2.178	2.237	2.157	2.200
Mainchain	2.140	2.202	2.162	2.205	2.165	2.226	2.142	2.187
Sidechain	2.167	2.216	2.160	2.202	2.190	2.247	2.170	2.212
Backbone	2.137	2.200	2.160	2.202	2.163	2.223	2.140	2.185
C alpha	2.141	2.204	2.165	2.207	2.167	2.227	2.144	2.189
Ligand	0.541	0.575	0.378	0.384	0.570	0.558	0.394	0.413
