# Supplementary material for: Ligand-Based and Structure-Based Investigation for Alzheimer's Disease from Traditional Chinese Medicine
Source: Evid Based Complement Alternat Med. 2014 May 8;2014:364819. doi: 10.1155/2014/364819 (PMC4034731; doi:10.1155/2014/364819)
Supplement: Supplementary file 1 — Table S1. Descriptions of representative descriptors associated with bioactivity determined by GFA. Table S2. H-bond interactions of FKBP52 with TCM candidates and Tacrolimus in MD simulation. Figure S1. Details about torsion angles of control and TCM candidates in FKBP52 complex. (A) Tacrolimus, (B) Daphnetoxin, (C) 20-O-(2´E,4´E-decadienoyl)ingenol, and (D) Lythrancine II. (gray(X):original torsion anglesred(Y): if X<0,Y=X+360, blue(Z)=Y(i+1)-Y) Figure S2. Distance matrices depicting the smallest distance between residue pairs. (A) Tacrolimus, (B) Daphnetoxin, (C) 20-O-(2´E,4´E-decadienoyl)ingenol, and (D) Lythrancine II. [file 364819.f1.docx]

**Table S1.** Descriptions of representative descriptors associated with bioactivity determined by GFA.

| Descriptor | Description |
| --- | --- |
| *ES_Count_aaCH* | Sum of electrotopological count for aromatic bond CH. |
| *ES_Count_sOH* | Sum of electrotopological count for single bond OH. |
| *ES_Sum_aaCH* | Sum of electrotopological sum for aromatic bond CH. |
| *ES_Sum_sssN* | Sum of electrotopological sum for single bond N. |
| *Num_Rings6* | Number of rings of size 6. |
| *Molecular_PolarSurfaceArea* | Calculates the polar surface area for each molecule. |
| *CHI_2* | Connectivity indices derived from the 2D-topology of the molecule. |
| *Jurs_TPSA* | Calculates total polar surface area by the sum of solvent-accessible surface areas of atoms with absolute value of partial charges greater or equal than 0.2 |
| *Minimized_Energy* | Energy of molecule following a fast minimization procedure using clean force field. |
| *Shadow_Ylength* | Geometric descriptor that describes the length of the molecule in the *y* dimension |

**Table S2.** H-bond interactions of FKBP52 with TCM candidates and Tacrolimus in MD simulation.

| **Ligand** | **H-bond** | **Ligand Atom** | **Amino acid** | **Distance (Å)** | | | **H-bond occupancy** |
| --- | --- | --- | --- | --- | --- | --- | --- |
|  |  |  |  | **Max.** | **Min.** | **Average** |  |
| Tacrolimus | 1 | H114 | Gly84:O | 14.93 | 1.64 | 8.83 | 5.49% |
|  | 2 | H114 | Glu110:O | 11.83 | 1.60 | 4.47 | 9.69% |
|  | 3 | H114 | Ala112:O | 11.09 | 1.55 | 4.95 | 2.20% |
|  | 4 | O10 | Tyr113:HH | 18.46 | 1.83 | 12.08 | 0.30% |
|  | 5 | O12 | Tyr113:HH | 18.86 | 1.65 | 12.94 | 1.60% |
|  | 6 | O10 | Ser118:HG1 | 14.36 | 1.60 | 8.17 | 12.49% |
|  | 7 | O12 | Ser118:HG1 | 14.70 | 1.61 | 8.98 | 3.40% |
| Daphnetoxin | 1 | H49 | Tyr57:OH | 9.33 | 1.74 | 5.44 | 8.49% |
|  | 2 | H52 | Glu85:O | 16.25 | 1.55 | 6.67 | 2.40% |
|  | 3 | O23 | Ile87:HN | 15.75 | 1.84 | 6.69 | 8.79% |
|  | 4 | O16 | Tyr113:HH | 8.08 | 1.48 | 3.74 | 31.47% |
|  | 5 | O18 | Tyr113:HH | 8.93 | 1.65 | 4.44 | 2.00% |
|  | 6 | H52 | Tyr113:OH | 13.40 | 1.79 | 7.35 | 9.89% |
| 20-O-(2´*E*,4´*E*-decadienoyl)ingenol | 1 | H59 | Tyr57:OH | 10.59 | 1.86 | 6.16 | 2.50% |
|  | 2 | H59 | Asp68:OD1 | 12.35 | 1.64 | 6.01 | 20.78% |
|  | 3 | H59 | Asp68:OD2 | 13.04 | 1.66 | 6.04 | 2.40% |
|  | 4 | O21 | Tyr113:HH | 10.38 | 1.68 | 6.63 | 1.80% |
|  | 5 | H60 | Tyr113:OH | 11.24 | 1.82 | 6.92 | 0.60% |
|  | 6 | O21 | Lys121:HZ3 | 16.74 | 1.74 | 6.06 | 9.09% |
|  | 7 | O22 | Lys121:HZ3 | 17.22 | 1.69 | 7.55 | 8.99% |
| Lythrancine II | 1 | H74 | Tyr57:OH | 11.15 | 1.78 | 4.89 | 1.60% |
|  | 2 | H74 | Asp68:OD1 | 11.91 | 1.53 | 4.25 | 13.49% |
|  | 3 | H74 | Asp68:OD2 | 13.37 | 1.65 | 4.30 | 15.98% |
|  | 4 | O36 | Ile87:HN | 17.31 | 1.84 | 4.45 | 35.66% |
|  | 5 | O19 | Tyr113:HH | 15.92 | 1.62 | 3.80 | 9.29% |
|  | 6 | O36 | Tyr113:HH | 17.39 | 1.63 | 5.69 | 3.50% |
|  | 7 | H51 | Ser118:OG | 18.53 | 1.64 | 5.54 | 19.98% |

**H-bond occupancy cutoff: 2.5** Å

**Supplementary Figure Legends**

**
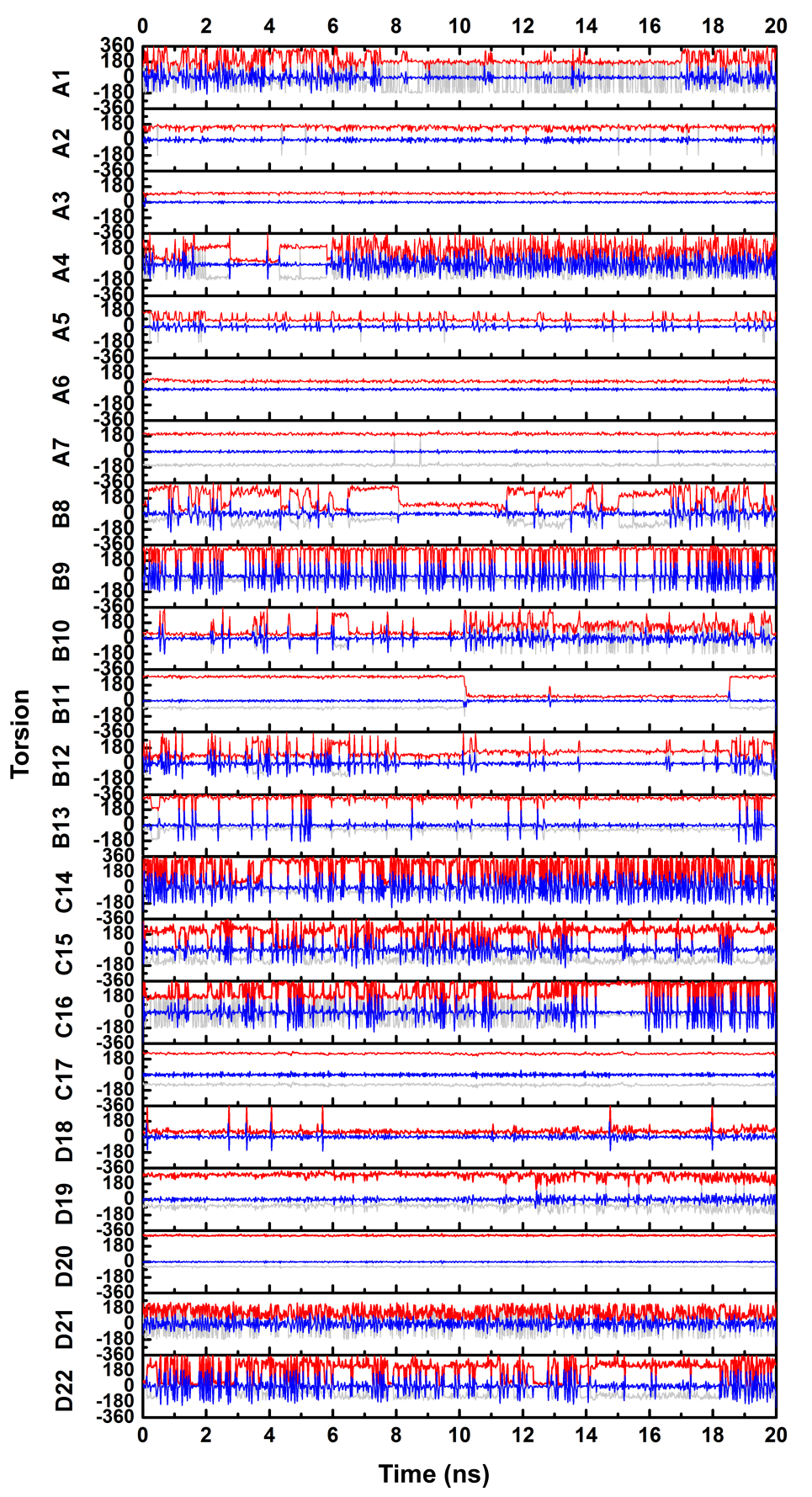
**

**Figure S1.** Details about torsion angles of control and TCM candidates in FKBP52 complex. (A) Tacrolimus, (B) Daphnetoxin, (C) 20-O-(2´E,4´E-decadienoyl)ingenol, and (D) Lythrancine II. (gray(X):original torsion angles，red(Y): if X<0,Y=X+360, blue(Z)=Y(i+1)-Y)


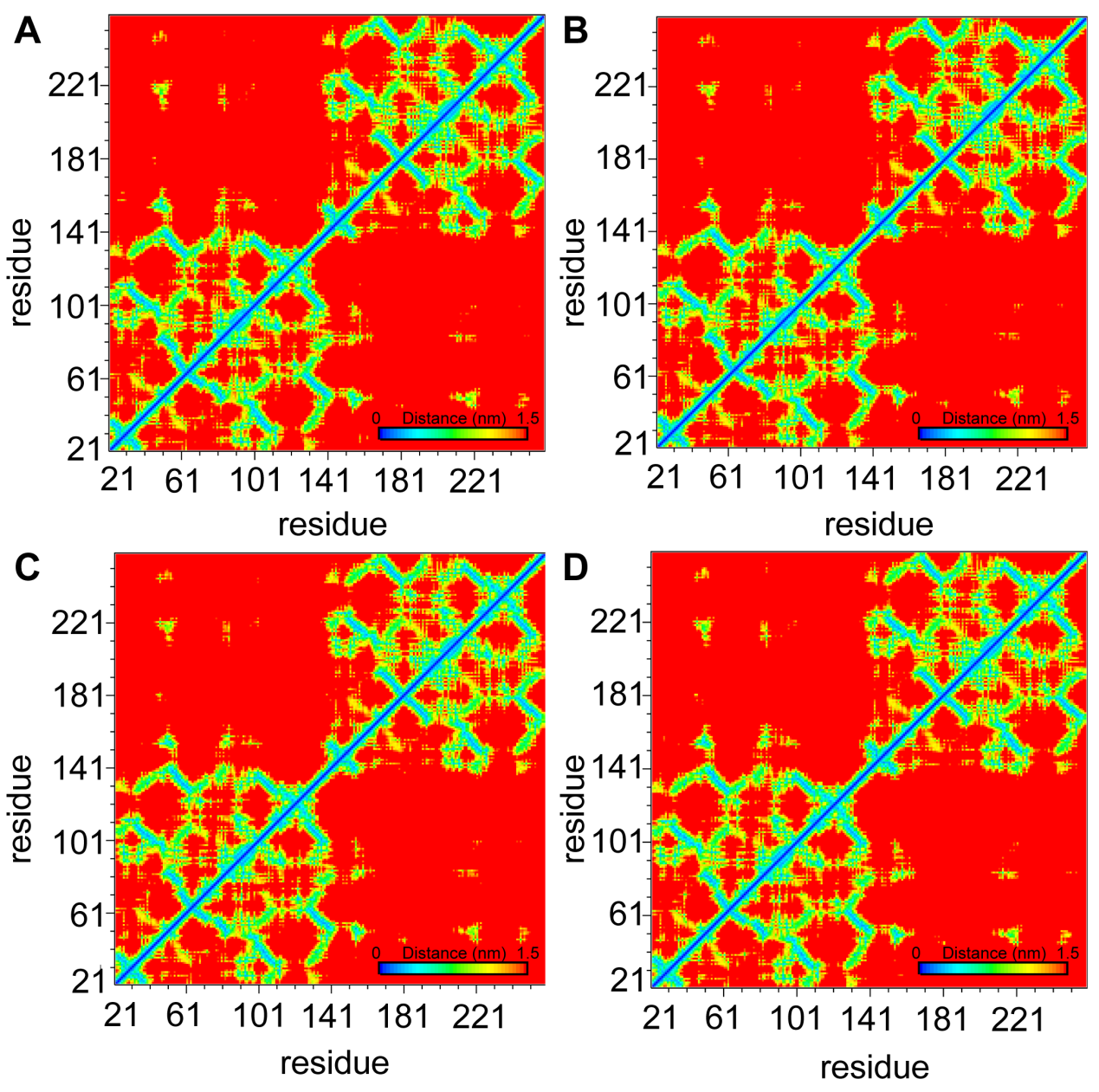


**Figure S2.** Distance matrices depicting the smallest distance between residue pairs. (A) Tacrolimus, (B) Daphnetoxin, (C) 20-O-(2´*E*,4´*E*-decadienoyl)ingenol, and (D) Lythrancine II.
